# Tobacco Product Use Among U.S. Middle and High School Students — National Youth Tobacco Survey, 2023

**DOI:** 10.15585/mmwr.mm7244a1

**Published:** 2023-11-03

**Authors:** Jan Birdsey, Monica Cornelius, Ahmed Jamal, Eunice Park-Lee, Maria R. Cooper, Jia Wang, Michael D. Sawdey, Karen A. Cullen, Linda Neff

**Affiliations:** ^1^Office on Smoking and Health, National Center for Chronic Disease Prevention and Health Promotion, CDC; ^2^Center for Tobacco Products, Food and Drug Administration, Silver Spring, Maryland.

**Summary**

**What is already known about this topic?**

Use of tobacco products in any form by youths is unsafe.

**What is added by this report?**

In 2023, 10.0% of middle and high school students reported current tobacco product use. From 2022 to 2023, current e-cigarette use among high school students declined from 14.1% to 10.0%. E-cigarettes remained the most commonly used tobacco product among youths. Among middle school and high school students who currently use e-cigarettes, 25.2% used e-cigarettes daily, and 89.4% used flavored e-cigarettes.

**What are the implications for public health?**

Tobacco use declined among high school students; however, sustained public health monitoring with implementation of evidence-based tobacco control strategies, including effective youth interventions, media campaigns, Food and Drug Administration regulations, and other proven tobacco prevention policies might further reduce youth tobacco product use.

## Abstract

Tobacco product use during adolescence increases the risk for lifelong nicotine addiction and adverse health consequences. CDC and the Food and Drug Administration analyzed data from the 2023 National Youth Tobacco Survey to assess tobacco product use patterns among U.S. middle school (grades 6–8) and high school (grades 9–12) students. In 2023, 10.0% of middle and high school students (2.80 million) reported current (i.e., past 30-day) use of any tobacco product. Current use of any tobacco product by high school students declined by an estimated 540,000, from 2.51 million in 2022 to 1.97 million in 2023. From 2022 to 2023, current e-cigarette use among high school students declined from 14.1% to 10.0%. Among middle and high school students, e-cigarette products were the most used tobacco product in 2023 (7.7%; 2.13 million), followed by cigarettes (1.6%), cigars (1.6%), nicotine pouches (1.5%), smokeless tobacco (1.2%), other oral nicotine products (1.2%), hookahs (1.1%), heated tobacco products (1.0%), and pipe tobacco (0.5%). Among students who had ever used an e-cigarette, 46.7% reported current use. In 2023, among students reporting current e-cigarette use, 89.4% used flavored products and 25.2% used an e-cigarette daily; the most commonly reported brands were Elf Bar, Esco Bars, Vuse, JUUL, and Mr. Fog. Given the number of middle and high school students that use tobacco products, sustained efforts to prevent initiation of tobacco product use among young persons and strategies to help young tobacco users quit are critical to reducing U.S. youth tobacco product use.

## Introduction

Commercial tobacco use* among U.S. youths can lead to lifelong nicotine addiction (*1*) and subsequent disability, disease, and death (*2*). This report presents findings from the 2023 National Youth Tobacco Survey (NYTS) and describes the prevalence of ever use (i.e., ever having used, even once or twice) and current use of nine tobacco product types, flavored tobacco products, and e-cigarette use behaviors among U.S. middle and high school students. In addition, 2023 NYTS results are compared with those reported for 2022 NYTS data (*3*).

## Methods

### Data Collection

The NYTS is a cross-sectional, school-based, self-administered web-based survey of U.S. middle and high school students. A stratified, three-stage cluster sampling procedure was used to generate a nationally representative sample of U.S. students attending private or public middle (grades 6–8) and high (grades 9–12) schools. In 2023, data were collected during March 9–June 16; a total of 22,069 students from 179 schools participated, with an overall response rate of 30.5%. 

### Data Analysis

National weighted prevalence estimates, 95% CIs, and population totals^†^ were calculated for ever use (i.e., ever having used, even once or twice) and current use (i.e., use on ≥1 days during the past 30 days) of nine commercial tobacco products^§^ (e-cigarettes, cigarettes, cigars, smokeless tobacco, nicotine pouches,^¶^ hookahs, pipe tobacco, heated tobacco products,** and other oral nicotine products) by student characteristics. Three composite measures were also reported for use of any tobacco product,^††^ any combustible tobacco product,^§§^ and multiple tobacco products.^¶¶^ Current e-cigarette use (i.e., use on ≥1 day during the past 30 days) was reported by frequency of use, device type,*** brand,^†††^ and flavor.^§§§^ Changes in current-use prevalence since 2022 were estimated using t-tests; details of the 2022 NYTS data collection and estimates have been published previously (*3*). P-values <0.05 were considered statistically significant. Analyses were conducted using SAS-callable SUDAAN software (version 11.0.4; Research Triangle Institute). Estimates with an unweighted denominator <50 or a relative SE >30% were suppressed. This activity was reviewed by CDC, deemed not research, and was conducted consistent with applicable federal law and CDC policy.^¶¶¶^

## Results

### Tobacco Product Use by Population

In 2023, 22.2% of U.S. middle and high school students reported ever using any tobacco product, corresponding to 6.21 million persons (Table 1); 10.0% of students reported current use of any tobacco product, corresponding to 2.80 million persons (Table 2). Overall, current use of any tobacco product was reported by 11.2% of female, 8.9% of male, 12.6% of non-Hispanic multiracial (multiracial), 11.7% of Hispanic or Latino (Hispanic), 9.5% of non-Hispanic White (White), 9.3% of non-Hispanic Black or African American (Black), and 8.0% of non-Hispanic American Indian or Alaska Native (AI/AN) students.**** Current use of any combustible tobacco product was reported by 4.7% of Black, 3.9% of Hispanic, 3.7% of multiracial, and 2.7% of White and AI/AN students.

**TABLE 1:** Percentage of middle and high school students who reported ever using tobacco products,* by product, overall and by school level, sex, and race and ethnicity — National Youth Tobacco Survey, United States, 2023

Tobacco product	% (95% CI)										Total estimated weighted no.^§^
	Sex		Race and ethnicity^†^							Total	
	Female	Male	AI/AN, NH	Asian, NH	Black or African American, NH	White, NH	Hispanic or Latino	Multiracial, NH			
**Overall**											
Any tobacco product^¶^	23.7 (21.5–26.0)	20.8 (18.9–22.8)	22.7 (16.8–30.0)	12.1 (6.5–21.5)	20.1 (17.7–22.6)	23.1 (20.2–26.2)	23.8 (22.2–25.4)		27.9 (22.5–33.9)	**22.2 (20.5–23.9)**	**6,210,000**
E-cigarettes	19.4 (17.5–21.5)	14.7 (13.2–16.3)	15.4 (10.7–21.8)	—**	12.9 (11.1–14.8)	18.4 (15.9–21.1)	18.2 (16.3–20.2)		20.8 (15.9–26.8)	**17.0 (15.6–18.5)**	**4,750,000**
Cigarettes	7.0 (6.0–8.1)	6.5 (5.4–7.7)	9.5 (5.6–15.5)	—	4.1 (2.9–5.8)	7.5 (6.3–8.9)	7.4 (5.9–9.2)		8.7 (6.0–12.4)	**6.7 (6.0–7.6)**	**1,840,000**
Cigars^††^	3.8 (2.9–4.8)	5.8 (4.8–7.0)	—	—	4.7 (3.4–6.4)	5.2 (4.1–6.6)	4.7 (4.0–5.5)		6.9 (4.8–9.8)	**4.8 (4.0–5.6)**	**1,300,000**
Hookahs	3.4 (2.4–4.8)	2.7 (1.9–3.8)	—	—	4.5 (2.7–7.2)	2.5 (1.7–3.5)	3.5 (2.7–4.5)		3.6 (2.4–5.2)	**3.0 (2.4–3.9)**	**820,000**
Smokeless tobacco (composite)^††^	2.2 (1.7–2.9)	3.7 (2.8–4.8)	—	—	1.3 (0.8–2.1)	3.4 (2.5–4.6)	2.9 (2.2–3.8)		5.0 (3.3–7.5)	**3.0 (2.4–3.6)**	**800,000**
Other oral nicotine products^††^	2.7 (2.1–3.4)	3.2 (2.6–4.1)	4.9 (2.8–8.5)	—	1.7 (1.1–2.6)	3.2 (2.4–4.1)	3.5 (2.7–4.6)		4.2 (2.4–7.2)	**3.0 (2.5–3.5)**	**800,000**
Nicotine pouches	1.7 (1.2–2.4)	3.0 (2.2–4.1)	—	—	—	3.0 (2.3–3.9)	2.0 (1.2–3.2)		—	**2.3 (1.8–3.0)**	**580,000**
Pipe tobacco	1.5 (1.1–2.0)	1.9 (1.4–2.5)	—	—	—	1.8 (1.3–2.5)	2.0 (1.5–2.7)		2.3 (1.3–3.9)	**1.7 (1.4–2.0)**	**440,000**
Heated tobacco products	1.5 (1.1–2.0)	1.5 (1.0–2.1)	—	—	1.7 (1.0–2.9)	1.4 (0.9–2.0)	1.8 (1.3–2.4)		1.6 (0.9–3.0)	**1.5 (1.1–2.0)**	**370,000**
Any combustible tobacco product^§§^	10.9 (9.3–12.8)	11.6 (10.1–13.2)	11.1 (7.0–17.1)	4.4 (2.4–7.8)	11.2 (8.5–14.7)	11.6 (9.7–13.7)	12.0 (10.4–13.8)		14.4 (11.0–18.5)	**11.2 (9.9–12.7)**	**3,090,000**
Multiple tobacco products^¶¶^	10.1 (8.7–11.8)	9.6 (8.4–10.9)	11.0 (7.2–16.3)	3.6 (2.1–6.0)	7.3 (5.5–9.7)	10.8 (9.1–12.8)	10.3 (8.9–11.8)		13.3 (10.1–17.3)	**9.8 (8.7–11.1)**	**2,750,000**
**High school students (grades 9–12)**											
Any tobacco product^¶^	30.1 (26.9–33.5)	25.9 (23.5–28.5)	29.0 (19.1–41.5)	—	21.8 (18.8–25.2)	31.4 (28.0–34.9)	27.3 (24.8–29.8)		35.1 (27.3–43.7)	**27.9 (25.8–30.2)**	**4,390,000**
E-cigarettes	26.0 (23.2–29.0)	19.5 (17.6–21.5)	20.3 (12.5–31.2)	—	14.7 (11.7–18.2)	26.0 (23.0–29.2)	22.3 (20.0–24.9)		27.5 (20.9–35.3)	**22.6 (20.9–24.5)**	**3,550,000**
Cigarettes	8.8 (7.3–10.6)	8.3 (7.0–9.7)	—	—	3.0 (1.8–5.0)	10.5 (9.0–12.1)	8.8 (6.9–11.1)		10.5 (6.8–15.7)	**8.5 (7.7–9.5)**	**1,310,000**
Cigars^††^	4.8 (3.6–6.4)	7.9 (6.3–9.9)	—	—	4.8 (3.2–7.1)	7.8 (6.1–10.0)	5.4 (4.4–6.6)		9.6 (6.4–14.0)	**6.4 (5.3–7.7)**	**980,000**
Hookahs	4.0 (2.7–5.9)	3.5 (2.3–5.4)	—	—	—	3.6 (2.5–5.3)	3.9 (2.7–5.5)		3.3 (1.9–5.8)	**3.7 (2.8–5.1)**	**560,000**
Smokeless tobacco (composite)^††^	2.2 (1.5–3.2)	4.3 (3.3–5.7)	—	—	—	3.8 (2.8–5.1)	2.9 (2.1–4.0)		6.9 (4.1–11.4)	**3.3 (2.6–4.1)**	**500,000**
Other oral nicotine products^††^	2.8 (2.0–4.0)	4.0 (3.1–5.3)	—	—	1.6 (0.9–2.7)	4.1 (3.0–5.4)	3.8 (3.0–4.8)		—	**3.5 (2.8–4.2)**	**520,000**
Nicotine pouches	2.0 (1.4–2.9)	4.1 (3.0–5.6)	—	—	—	4.5 (3.5–5.7)	1.8 (1.1–2.8)		—	**3.1 (2.4–4.0)**	**430,000**
Pipe tobacco	1.7 (1.2–2.5)	2.4 (1.8–3.2)	—	—	—	2.7 (2.0–3.5)	2.2 (1.5–3.2)		3.3 (2.0–5.5)	**2.1 (1.7–2.5)**	**310,000**
Heated tobacco products	1.7 (1.2–2.5)	1.6 (1.0–2.4)	—	—	—	1.8 (1.2–2.8)	1.5 (0.9–2.3)		—	**1.6 (1.2–2.3)**	**230,000**
Any combustible tobacco product^§§^	13.6 (11.3–16.2)	14.9 (13.0–16.9)	—	—	10.7 (8.2–14.0)	16.4 (14.1–19.1)	13.8 (11.7–16.3)		17.5 (12.6–23.7)	**14.2 (12.6–16.1)**	**2,190,000**
Multiple tobacco products^¶¶^	12.8 (10.5–15.4)	12.6 (11.1–14.2)	14.2 (8.0–24.0)	4.6 (2.5–8.3)	7.1 (4.9–10.1)	15.4 (13.1–18.1)	11.7 (10.0–13.6)		17.1 (12.2–23.3)	**12.7 (11.1–14.4)**	**1,990,000**
**Middle school students (grades 6–8)**											
Any tobacco product^¶^	15.4 (12.9–18.3)	13.8 (11.3–16.6)	15.3 (9.7–23.2)	—	17.8 (12.9–24.0)	12.3 (10.0–14.9)	18.7 (16.5–21.1)		17.6 (13.0–23.6)	**14.7 (12.5–17.1)**	**1,780,000**
E-cigarettes	11.0 (9.1–13.3)	8.2 (6.9–9.8)	—	—	10.6 (8.5–13.1)	8.4 (6.8–10.3)	12.3 (10.5–14.4)		11.3 (6.3–19.5)	**9.7 (8.3–11.3)**	**1,170,000**
Cigarettes	4.6 (3.6–5.9)	4.0 (2.7–5.9)	—	—	5.5 (3.9–7.8)	3.5 (2.5–5.1)	5.3 (3.8–7.2)		—	**4.3 (3.3–5.5)**	**510,000**
Cigars^††^	2.4 (1.6–3.6)	2.9 (2.0–4.2)	—	—	4.6 (2.8–7.4)	1.7 (1.1–2.6)	3.5 (2.3–5.3)		—	**2.6 (1.9–3.7)**	**310,000**
Hookahs	—	1.7 (1.2–2.3)	—	—	—	0.9 (0.5–1.6)	2.9 (2.1–4.0)		—	**2.1 (1.4–3.2)**	**240,000**
Smokeless tobacco (composite)^††^	2.3 (1.6–3.1)	2.7 (1.8–4.0)	—	—	—	2.9 (1.9–4.4)	2.5 (1.6–3.9)		—	**2.4 (1.8–3.3)**	**290,000**
Other oral nicotine products^††^	2.4 (1.8–3.2)	2.1 (1.6–2.7)	—	—	—	2.0 (1.4–2.9)	2.9 (1.8–4.4)		2.9 (1.6–5.2)	**2.2 (1.8–2.7)**	**260,000**
Nicotine pouches	—	—	—	—	—	1.0 (0.6–1.8)	—		—	**—**	**—**
Pipe tobacco	1.1 (0.6–2.0)	1.1 (0.6–2.0)	—	—	—	—	1.7 (1.2–2.4)		—	**1.1 (0.7–1.6)**	**120,000**
Heated tobacco products	1.2 (0.7–1.9)	—	—	—	—	0.8 (0.5–1.5)	2.1 (1.6–2.8)		—	**1.2 (0.8–1.8)**	**130,000**
Any combustible tobacco product^§§^	7.5 (5.7–10.0)	7.2 (5.1–9.9)	6.6 (3.6–11.7)	—	11.9 (7.0–19.4)	5.3 (3.8–7.3)	9.3 (7.3–11.7)		9.8 (6.4–14.8)	**7.3 (5.6–9.4)**	**870,000**
Multiple tobacco products^¶¶^	6.7 (5.3–8.6)	5.5 (4.2–7.2)	—	—	7.6 (4.7–12.2)	4.7 (3.5–6.2)	8.0 (6.0–10.6)		7.9 (5.3–11.6)	**6.1 (4.9–7.5)**	**740,000**

**Abbreviations**: AI/AN = American Indian or Alaska Native; NH = non-Hispanic.

* Ever use is defined as ever having used the product, even once or twice. Because of missing data on the ever use questions, denominators for each tobacco product might be different. For each question, response options were “yes” or “no.”

^†^ Hispanic or Latino persons could be of any race. Estimates among NH Native Hawaiian or other Pacific Islander students, overall and by school level, were statistically unreliable for all measures and are not presented in this table.

^§^ Estimated weighted total number of ever tobacco product users was rounded down to the nearest 10,000 persons. Overall estimates were reported based on 22,069 U.S. middle and high school students. School level was determined by reported grade level: high school (grades 9–12; n = 10,879) and middle school (grades 6–8; n = 11,067). The sum of subgroup estimates might not sum to overall population estimates because of rounding or exclusion of students who did not report sex, race and ethnicity, or grade level.

^¶^ Any tobacco product use is defined as ever use of one or more of the following tobacco products: e-cigarettes, cigars, cigarettes, smokeless tobacco (composite), hookahs, nicotine pouches, heated tobacco products, pipe tobacco, bidis (small brown cigarettes wrapped in a leaf), or other oral nicotine products.

** Dashes indicate that data were statistically unreliable because of an unweighted denominator <50 or a relative SE >30%.

^††^ Cigars were defined as cigars, cigarillos, or little cigars. Smokeless tobacco (composite) was defined as chewing tobacco, snuff, dip, or snus. Other oral nicotine products were defined as lozenges, discs, tablets, gums, dissolvable tobacco products, and other products. In 2023, dissolvable tobacco products were reclassified from smokeless tobacco to other oral nicotine products.

^§§^ Any combustible tobacco product use was defined as ever use of one or more of the following tobacco products: cigarettes, cigars, hookahs, pipe tobacco, or bidis.

^¶¶^ Multiple tobacco product use was defined as ever use of two or more of the following tobacco products: e-cigarettes, cigars, cigarettes, smokeless tobacco (composite), hookahs, nicotine pouches, heated tobacco products, pipe tobacco, bidis, or other oral nicotine products.

**TABLE 2:** Percentage of middle and high school students who reported current (past 30-day) tobacco product use, by product,* overall and by school level, sex, and race and ethnicity — National Youth Tobacco Survey, United States, 2023

Tobacco product	% (95% CI)								Total estimated weighted no.^§^
	Sex		Race and ethnicity^†^					Total	
	Female	Male	AI/AN, NH	Black or African American, NH	White, NH	Hispanic or Latino	Multiracial, NH		
**Overall**									
Any tobacco product^¶^	11.2 (9.5–13.1)	8.9 (7.7–10.3)	8.0 (4.7–13.2)	9.3 (7.5–11.3)	9.5 (7.7–11.6)	11.7 (10.1–13.4)	12.6 (8.8–17.7)	**10.0 (8.9–11.2)**	**2,800,000**
E-cigarettes	9.3 (8.1–10.8)	6.1 (5.0–7.4)	5.9 (3.4–10.0)	5.6 (4.5–7.1)	7.7 (6.3–9.4)	8.5 (7.4–9.8)	10.2 (6.8–15.1)	**7.7 (6.8–8.6)**	**2,130,000**
Cigarettes	1.4 (1.0–1.9)	1.8 (1.4–2.4)	—**	—	1.6 (1.1–2.3)	2.1 (1.5–3.1)	1.6 (1.0–2.8)	**1.6 (1.2–2.1)**	**430,000**
Cigars	1.3 (0.9–2.0)	1.8 (1.4–2.3)	—	2.3 (1.4–3.8)	1.0 (0.7–1.4)	2.2 (1.7–2.8)	—	**1.6 (1.2–2.0)**	**420,000**
Nicotine pouches	0.8 (0.5–1.3)	2.1 (1.5–3.0)	—	—	1.4 (0.9–2.2)	1.9 (1.1–3.3)	—	**1.5 (1.0–2.1)**	**400,000**
Smokeless tobacco (composite)^††^	—	1.6 (1.1–2.3)	—	—	1.2 (0.7–1.8)	1.6 (1.1–2.4)	—	**1.2 (0.9–1.7)**	**330,000**
Other oral nicotine products	1.1 (0.9–1.4)	1.2 (0.9–1.7)	0.5 (0.3–0.8)	—	1.2 (0.9–1.5)	1.5 (1.1–2.0)	—	**1.2 (1.0–1.4)**	**310,000**
Hookahs	1.3 (0.8–2.1)	0.9 (0.6–1.3)	—	—	0.7 (0.4–1.1)	1.3 (1.0–1.7)	1.3 (0.7–2.4)	**1.1 (0.8–1.5)**	**290,000**
Heated tobacco products	0.7 (0.5–1.0)	1.2 (0.8–1.9)	—	1.0 (0.5–1.7)	0.7 (0.4–1.2)	1.5 (1.0–2.2)	—	**1.0 (0.7–1.3)**	**260,000**
Pipe tobacco	0.5 (0.3–0.7)	0.6 (0.4–0.9)	—	—	0.5 (0.3–0.8)	0.9 (0.5–1.4)	—	**0.5 (0.4–0.7)**	**130,000**
Any combustible tobacco product^§§^	3.3 (2.6–4.1)	3.5 (2.9–4.2)	2.7 (1.5–4.9)	4.7 (3.1–7.0)	2.7 (2.2–3.5)	3.9 (3.1–4.8)	3.7 (2.4–5.5)	**3.4 (2.9–4.0)**	**920,000**
Multiple tobacco products^¶¶^	3.4 (2.7–4.2)	3.4 (2.7–4.2)	2.0 (1.1–3.5)	3.2 (1.8–5.5)	3.1 (2.4–4.0)	3.9 (3.4–4.5)	4.1 (2.6–6.5)	**3.4 (2.9–3.9)**	**940,000**
**High school students (grades 9–12)**									
Any tobacco product^¶^	14.1 (11.6–17.0)	11.2 (9.4–13.1)	—	9.8 (7.7–12.5)	13.6 (11.2–16.5)	12.4 (10.6–14.4)	17.2 (11.3–25.3)	**12.6 (11.1–14.3)**	**1,970,000**
E-cigarettes	12.2 (10.3–14.5)	8.0 (6.3–10.0)	—	5.6 (4.2–7.4)	11.3 (9.2–13.7)	9.7 (8.0–11.8)	14.2 (9.0–21.8)	**10.0 (8.8–11.4)**	**1,560,000**
Cigarettes	1.5 (1.0–2.2)	2.3 (1.8–2.9)	—	—	2.2 (1.4–3.4)	2.2 (1.6–3.0)	—	**1.9 (1.5–2.4)**	**290,000**
Cigars	1.4 (0.8–2.3)	2.3 (1.7–3.0)	—	1.9 (1.2–3.0)	1.4 (0.9–2.2)	2.3 (1.6–3.3)	—	**1.8 (1.4–2.4)**	**280,000**
Nicotine pouches	—	2.6 (1.9–3.6)	—	—	2.2 (1.4–3.4)	1.6 (0.9–2.7)	—	**1.7 (1.2–2.5)**	**260,000**
Smokeless tobacco (composite)^††^	—	2.1 (1.4–3.0)	—	—	1.7 (1.1–2.6)	1.7 (1.1–2.7)	—	**1.5 (1.1–2.2)**	**230,000**
Other oral nicotine products	0.9 (0.7–1.3)	1.5 (1.0–2.2)	—	—	1.3 (1.0–1.8)	1.6 (1.1–2.2)	—	**1.2 (1.0–1.6)**	**180,000**
Hookahs	1.4 (0.8–2.4)	0.9 (0.6–1.5)	—	—	—	1.0 (0.6–1.6)	—	**1.1 (0.8–1.6)**	**170,000**
Heated tobacco products	0.7 (0.4–1.2)	1.4 (0.8–2.5)	—	—	—	1.6 (0.9–2.7)	—	**1.0 (0.7–1.6)**	**150,000**
Pipe tobacco	0.5 (0.3–0.9)	0.7 (0.4–1.2)	—	—	0.6 (0.4–1.0)	_	—	**0.6 (0.4–0.9)**	**90,000**
Any combustible tobacco product^§§^	3.6 (2.7–4.7)	4.3 (3.6–5.2)	—	4.5 (3.2–6.2)	3.8 (3.0–5.0)	3.8 (2.8–5.0)	5.3 (3.3–8.6)	**3.9 (3.4–4.6)**	**600,000**
Multiple tobacco products^¶¶^	3.5 (2.7–4.7)	4.3 (3.4–5.5)	—	—	4.3 (3.2–5.7)	3.9 (3.0–5.1)	6.1 (3.6–10.2)	**3.9 (3.3–4.7)**	**610,000**
**Middle school students (grades 6–8)**									
Any tobacco product^¶^	7.5 (5.9–9.4)	5.7 (4.1–8.0)	—	8.5 (5.8–12.4)	4.1 (3.2–5.2)	10.3 (7.5–14.0)	6.0 (3.4–10.5)	**6.6 (5.1–8.5)**	**800,000**
E-cigarettes	5.6 (4.5–7.1)	3.5 (2.5–4.8)	—	5.7 (3.9–8.2)	3.1 (2.2–4.2)	6.6 (5.3–8.2)	—	**4.6 (3.6–5.8)**	**550,000**
Cigarettes	1.1 (0.7–1.9)	—	—	—	0.8 (0.4–1.4)	—	—	**1.1 (0.6–1.9)**	**120,000**
Cigars	1.2 (0.7–2.2)	1.0 (0.6–1.8)	—	—	—	1.8 (1.1–3.1)	—	**1.1 (0.7–1.8)**	**130,000**
Nicotine pouches	—	—	—	—	0.5 (0.3–0.8)	—	—	**—**	**—**
Smokeless tobacco (composite)^††^	0.6 (0.4–1.0)	—	—	—	—	—	—	**0.7 (0.5–1.2)**	**80,000**
Other oral nicotine products	1.3 (0.9–1.8)	0.8 (0.6–1.3)	—	—	1.0 (0.6–1.6)	1.3 (0.8–2.0)	—	**1.1 (0.8–1.4)**	**120,000**
Hookahs	—	0.8 (0.5–1.5)	—	—	0.4 (0.2–0.7)	1.8 (1.1–2.9)	—	**1.0 (0.6–1.8)**	**120,000**
Heated tobacco products	0.8 (0.4–1.3)	—	—	—	—	1.3 (0.8–2.3)	—	**0.8 (0.5–1.4)**	**90,000**
Pipe tobacco	—	—	—	—	—	—	—	**0.4 (0.2–0.6)**	**40,000**
Any combustible tobacco product^§§^	2.8 (1.8–4.4)	2.3 (1.4–3.6)	—	—	1.3 (0.9–1.9)	3.7 (2.4–5.6)	—	**2.5 (1.7–3.8)**	**300,000**
Multiple tobacco products^¶¶^	3.1 (2.2–4.4)	2.0 (1.3–3.0)	—	—	1.5 (1.0–2.2)	3.5 (2.5–4.9)	—	**2.5 (1.8–3.5)**	**300,000**

**Abbreviations**: AI/AN = American Indian or Alaska Native; NH = non-Hispanic.

* Current use is defined as use on ≥1 days during the past 30 days for each product. Because of missing data on past 30-day use questions, denominators for each tobacco product might be different.

^†^ Hispanic or Latino persons could be of any race. Estimates among NH Asian and NH Native Hawaiian or other Pacific Islander students, overall and by school level, were statistically unreliable for all measures and are not presented in this table.

^§^ Estimated weighted total number of current tobacco product users was rounded down to the nearest 10,000 persons. Overall estimates were reported based on 22,069 U.S. middle and high school students. School level was determined by reported grade level: high school (grades 9–12; n = 10,879) and middle school (grades 6–8; n = 11,067). The sum of subgroup estimates might not sum to overall population estimates because of rounding or exclusion of students who did not report sex, race and ethnicity, or grade level.

^¶^ Any tobacco product use is defined as current use of one or more of the following tobacco products on ≥1 days during the past 30 days: e-cigarettes, cigars, cigarettes, smokeless tobacco (composite), hookahs, nicotine pouches, heated tobacco products, pipe tobacco, bidis (small brown cigarettes wrapped in a leaf), or other oral nicotine products.

** Dashes indicate that data were statistically unreliable because of an unweighted denominator <50 or a relative SE >30%.

^††^ Cigars were defined as cigars, cigarillos, or little cigars. Smokeless tobacco (composite) was defined as chewing tobacco, snuff, dip, or snus. Other oral nicotine products were defined as lozenges, discs, tablets, gums, dissolvable tobacco products, and other products. In 2023, dissolvable tobacco products were reclassified from smokeless tobacco to other oral nicotine products.

^§§^ Any combustible tobacco product use was defined as current use of one or more of the following tobacco products: cigarettes, cigars, hookahs, pipe tobacco, or bidis.

^¶¶^ Multiple tobacco product use was defined as current use of two or more of the following tobacco products: e-cigarettes, cigars, cigarettes, smokeless tobacco (composite), hookahs, nicotine pouches, heated tobacco products, pipe tobacco, bidis, or other oral nicotine products.

### Types of Tobacco Products Used

E-cigarettes were the most commonly reported currently used tobacco product among all students (7.7%) and both middle school (4.6%) and high school students (10.0%). Other currently used tobacco products included cigarettes (1.6%), cigars (1.6%), nicotine pouches (1.5%), smokeless tobacco (1.2%), other oral nicotine products (1.2%), hookahs (1.1%), heated tobacco products (1.0%), and pipe tobacco (0.5%). Among students who had ever used e-cigarettes, 46.7% reported current e-cigarette use.

### Characteristics of E-cigarette Use

Among students reporting current e-cigarette use, 25.2% reported using e-cigarettes daily. Frequent use (≥20 of the past 30 days) was reported by 34.7% of current e-cigarette users (Table 3). Disposable e-cigarettes were the most commonly reported device type used (60.7%), followed by prefilled or refillable pods or cartridges (16.1%), and tanks or mod systems (modifiable devices allowing users to customize the substances in the device) (5.9%). Among students who currently used e-cigarettes, Elf Bar was the most commonly reported brand (56.7%), followed by Esco Bars (21.6%), Vuse (20.7%), JUUL (16.5%), and Mr. Fog (13.6%).

**TABLE 3:** Percentage of middle and high school students reporting current (past 30-day) e-cigarette use,* overall by selected characteristics and school level — National Youth Tobacco Survey, United States, 2023

Characteristic	Overall		High school		Middle school	
	Estimated weighted no.^†^	% (95% CI)	Estimated weighted no.^†^	% (95% CI)	Estimated weighted no.^†^	% (95% CI)
**Among all students**	**2,130,000**	**7.7 (6.8–8.6)**	**1,560,000**	**10.0 (8.8–11.4)**	**550,000**	**4.6 (3.6–5.8)**
**Among current e-cigarette users**						
**Frequency of use during past 30 days**						
1–5 days	**980,000**	**46.1 (39.8–52.7)**	630,000	40.7 (33.1–48.7)	340,000	62.0 (55.7–67.9)
6–19 days	**400,000**	**19.1 (15.0–24.1)**	300,000	19.7 (14.1–26.8)	90,000	17.3 (12.2–24.0)
20–30 days	**740,000**	**34.7 (28.4–41.7)**	620,000	39.7 (31.3–48.6)	110,000	20.7 (14.6–28.6)
**Daily e-cigarette use^§^**	**530,000**	**25.2 (19.2–32.3)**	460,000	29.9 (22.1–39.1)	60,000	11.4 (7.5–17.0)
**Device type most often used** ^¶^						
Disposables	**1,240,000**	**60.7 (53.3–67.6)**	1,000,000	65.2 (56.3–73.1)	240,000	47.9 (39.5–56.5)
Prefilled or refillable pods or cartridges	**330,000**	**16.1 (12.2–21.0)**	240,000	16.0 (11.1–22.5)	80,000	16.7 (11.4–23.8)
Tanks or mod system	**120,000**	**5.9 (4.4–7.8)**	90,000	6.0 (4.3–8.4)	20,000	4.4 (2.5–7.5)
Don't know the type	**350,000**	**17.3 (12.7–23.1)**	190,000	12.8 (8.7–18.4)	150,000	31.1 (22.2–41.5)
Any brand**						
Elf Bar	**1,160,000**	**56.7 (50.6–62.6)**	900,000	59.1 (52.9–65.1)	260,000	50.0 (37.5–62.5)
Esco Bars	**440,000**	**21.6 (16.2–28.3)**	370,000	24.9 (18.1–33.1)	60,000	12.0 (6.9–20.2)
Vuse	**420,000**	**20.7 (16.4–25.9)**	330,000	22.2 (16.9–28.6)	80,000	16.3 (10.8–23.8)
JUUL	**330,000**	**16.5 (12.9–20.9)**	240,000	16.3 (11.8–22.1)	80,000	16.8 (11.4–24.1)
Mr. Fog	**280,000**	**13.6 (7.9–22.4)**	230,000	15.1 (8.2–26.3)	—^††^	—
SMOK (including NOVO)	**230,000**	**11.3 (6.3–19.5)**	—	—	30,000	6.7 (3.9–11.1)
Breeze	**230,000**	**11.6 (7.6–17.4)**	200,000	13.2 (8.0–21.2)	30,000	6.6 (4.1–10.5)
Kangvape (including Onee Stick)	**180,000**	**8.8 (6.6–11.7)**	130,000	8.7 (6.5–11.6)	—	—
Fume	**180,000**	**9.0 (6.4–12.6)**	140,000	9.2 (6.0–13.9)	40,000	8.2 (4.7–14.0)
NJOY	**150,000**	**7.5 (5.5–10.3)**	120,000	8.1 (5.6–11.7)	20,000	5.4 (3.1–9.2)
blu	**120,000**	**6.0 (4.4–8.3)**	70,000	5.2 (3.4–7.8)	40,000	8.1 (5.0–12.9)
HQD	**110,000**	**5.5 (3.4–8.5)**	80,000	5.7 (3.3–9.7)	—	—
Logic	**80,000**	**3.9 (2.5–6.1)**	50,000	3.7 (2.3–5.8)	—	—
Suorin (including Air Bar)	**70,000**	**3.8 (2.5–5.6)**	50,000	3.8 (2.3–6.3)	—	—
Lost Mary**^§§^**	**50,000**	**2.6 (1.4–4.8)**	40,000	3.3 (1.8–5.9)	—	—
Some other brand not listed	**350,000**	**17.3 (11.6–24.9)**	290,000	19.5 (12.4–29.2)	50,000	10.9 (6.0–19.0)
Not sure or don’t know the brand	**490,000**	**23.9 (19.3–29.2)**	300,000	19.8 (15.6–24.9)	180,000	35.4 (24.3–48.3)
**Usual brand^¶¶^**						
Elf Bar	**630,000**	**31.1 (26.2–36.4)**	460,000	30.2 (24.8–36.2)	170,000	33.9 (24.6–44.7)
Vuse	**170,000**	**8.7 (5.8–12.9)**	150,000	10.0 (6.4–15.3)	—	—
Esco Bars	**120,000**	**6.0 (3.4–10.4)**	110,000	7.7 (4.3–13.5)	—	—
JUUL	**70,000**	**3.4 (1.9–6.1)**	—	—	—	—
Mr. Fog	**—**	**—**	—	—	—	—
SMOK (including NOVO)	**—**	**—**	—	—	—	—
Breeze	**—**	**—**	—	—	—	—
Kangvape (including Onee Stick)	**—**	**—**	—	—	—	—
Fume	**—**	**—**	—	—	—	—
NJOY	**—**	**—**	—	—	—	—
blu	**—**	**—**	—	—	—	—
HQD	**—**	**—**	—	—	—	—
Logic	**—**	**—**	—	—	—	—
Suorin (including Air Bar)	**—**	**—**	—	—	—	—
Lost Mary	**—**	**—**	—	—	—	—
No usual brand	**90,000**	**4.4 (2.8–7.0)**	70,000	4.7 (2.8–7.5)	—	—
Some other brand not listed	**270,000**	**13.2 (7.8–21.5)**	230,000	15.4 (8.6–25.9)	—	—
Not sure or don’t know the brand	**400,000**	**19.8 (16.0–24.1)**	240,000	16.0 (12.5–20.4)	150,000	30.5 (22.1–40.4)
Flavored e-cigarette use***						
Any flavor other than tobacco-flavored or unflavored	**1,900,000**	**89.4 (86.2–91.9)**	1,410,000	90.3 (86.6–93.1)	480,000	87.1 (79.9–92.0)
Exclusive use of tobacco-flavored or unflavored	**110,000**	**5.6 (3.9–7.9)**	80,000	5.4 (3.4–8.4)	30,000	6.2 (3.4–11.0)
Unspecified	**100,000**	**5.0 (3.5–7.2)**	60,000	4.3 (2.8–6.6)	—	—
**Flavor type used among current e-cigarette users^†††^**						
Fruit	**1,280,000**	**63.4 (59.8–66.9)**	930,000	62.6 (57.9–67.0)	340,000	66.3 (59.5–72.5)
Candy, desserts, or other sweets	**700,000**	**35.0 (29.1–41.5)**	510,000	34.4 (27.5–42.1)	190,000	37.0 (28.6–46.4)
Mint	**560,000**	**27.8 (22.0–34.4)**	470,000	31.6 (24.2–40.1)	80,000	16.5 (11.6–22.9)
Menthol	**400,000**	**20.1 (15.5–25.8)**	340,000	23.3 (17.6–30.1)	50,000	10.4 (7.2–14.8)
Unflavored	**230,000**	**11.6 (8.8–15.1)**	160,000	10.9 (7.8–15.0)	60,000	13.2 (8.7–19.5)
Non-alcoholic drinks^§§§^	**220,000**	**11.3 (6.4–19.1)**	—	—	30,000	7.4 (4.1–13.0)
Alcoholic drinks^§§§^	**170,000**	**8.4 (5.5–12.7)**	130,000	9.0 (5.5–14.4)	—	—
Tobacco-flavored	**120,000**	**6.4 (4.5–9.0)**	70,000	5.3 (3.7–7.4)	—	—
Clove or spice ^§§§^	**120,000**	**6.0 (4.3–8.2)**	70,000	5.1 (3.3–7.7)	40,000	7.9 (4.6–13.1)
Chocolate	**90,000**	**4.9 (3.4–7.1)**	50,000	3.4 (1.9–6.2)	40,000	8.0 (4.3–14.3)
Some other flavor	**120,000**	**6.0 (4.2–8.5)**	60,000	4.6 (3.0–7.0)	50,000	10.0 (5.9–16.4)
**Use of any flavors that included the word “ice” or “iced” (such as “blueberry ice or strawberry ice”)^¶¶¶^**						
Yes	**1,100,000**	**57.9 (52.5–63.1)**	800,000	57.0 (51.3–62.6)	290,000	61.0 (52.8–68.5)
No	**560,000**	**29.5 (24.8–34.8)**	440,000	31.6 (26.1–37.7)	110,000	24.1 (18.2–31.1)
Don’t know	**230,000**	**12.6 (9.8–16.0)**	160,000	11.4 (8.3–15.5)	70,000	15.0 (10.2–21.5)
Use of any concept flavors with a name that did not describe a specific flavor (such as “solar,” “purple,” “jazz,” “island bash,” or “fusion”)****						
Yes	**300,000**	**16.1 (13.5–19.0)**	210,000	15.4 (12.2–19.3)	80,000	17.7 (12.3–24.7)
No	**1,110,000**	**58.5 (52.5–64.3)**	850,000	60.9 (52.8–68.4)	250,000	52.8 (45.5–60.0)
Don’t know	**480,000**	**25.4 (21.6–29.7)**	330,000	23.7 (19.0–29.2)	140,000	29.5 (22.6–37.5)

* Current (past 30-day) use of e-cigarettes was determined by asking, “During the past 30 days, on how many days did you use e-cigarettes?” Current use was defined as use on ≥1 days during the past 30 days.

^†^ Estimated total number of users was rounded down to the nearest 10,000 persons. The sum of subgroup estimates might not sum to overall population estimates because of rounding or exclusion of students who did not report grade level (n = 102), device type (n = 53), any brand (n = 54), usual brand (n = 61), flavor types used (n = 84), use of flavor including the word “ice” or “iced” (n = 136), or use of flavors without specific flavor descriptor (n = 143).

^§^ Daily e-cigarette use was defined as use on all 30 of the past 30 days.

^¶^ Device type was determined by the question, “Which of the following best describes the type of e-cigarette you have used in the past 30 days? If you have used more than one type, please think about the one you use most often.”

** All current e-cigarette users were asked, “During the past 30 days, what e-cigarette brands did you use? (Select one or more).” Those who selected “some other brand(s) not listed here” could provide a write-in response. Write-in responses corresponding to an original response option were recoded.

^††^ Data were statistically unreliable because of an unweighted denominator <50 or a relative SE >30%.

^§§^ Lost Mary was not included in the list of prespecified response options, but it was the most commonly provided write-in response for “some other brand(s) not listed here.”

^¶¶^ If a single brand was selected for the question, “During the past 30 days, what e-cigarette brands did you use (Select one or more),” it was reported as the respondent’s usual brand. Those who selected one or more brands were asked, “During the past 30 days, what brand of e-cigarettes did you usually use? (Choose only one answer).” Those who selected “some other brand(s) not listed here” could provide a write-in response. Write-in responses corresponding to an original response option were recoded.

*** All current e-cigarette users were asked, “In the past 30 days when you used e-cigarettes, what flavors did you use? (Select one or more)?” Those who provided no valid responses were defined as “Unspecified” flavored users.

^†††^ Flavor type was determined by response to the question, “In the past 30 days when you used e-cigarettes, what flavors did you use? (Select one or more).” Those who selected “some other flavor not listed here” could provide a write-in response; write-in responses corresponding to an original response option were recoded.

^§§§^ These flavor options provided examples: “Alcoholic drinks (such as wine, margarita, or other cocktails)”; “Non-alcoholic drinks (such as coffee, soda, lemonade, or other beverage)”; “Spice (such as cinnamon, vanilla, or clove).”

^¶¶¶^ Current e-cigarette users were asked, “Did any of the flavors you used in the past 30 days have names or descriptions that included the word ‘ice’ or ‘iced’ (for example, blueberry ice or strawberry ice)?” Those who reported using only unflavored e-cigarettes (n = 60) did not receive the question.

**** Current e-cigarette users were asked, “Did any of the flavors that you used in the past 30 days have a name that did not describe a specific flavor, such as ‘solar,’ ‘purple,’ ‘jazz,’ ‘island bash,’ ‘fusion,’ or some other word or phrase?” Those who reported using only “unflavored” e-cigarettes (n = 60) did not receive the question.

Among students reporting current e-cigarette use, 89.4% reported using a flavored product during the past 30 days, excluding those who only used tobacco-flavored or unflavored e-cigarettes (Table 3). Among students who currently used e-cigarettes, fruit- (63.4%) and candy- (35.0%) flavored categories were reported most commonly; 6.4% of students reported use of tobacco-flavored e-cigarettes. Among those who currently used disposable e-cigarettes, the top reported flavor categories were fruit (70.5%), candy (39.8%), mint (32.0%), menthol (18.7%), unflavored (7.8%), alcoholic drinks (7.2%), and tobacco-flavored (5.4%) (Supplementary Table 1, https://stacks.cdc.gov/view/cdc/134700). Among students reporting current use of any tobacco product, 86.9% used a flavored product, ranging from 40.4% of cigarette users (menthol) to 89.4% of e-cigarette users (Supplementary Table 2, https://stacks.cdc.gov/view/cdc/134701). Among students currently using tobacco products, use of products with “ice” or “iced”^††††^ included in the flavor name was reported by 57.9% of e-cigarette users, 25.9% of nicotine pouch users, and 22.6% of cigar users; use of concept flavors^§§§§^ was reported by 16.1% of e-cigarette users and 13.4% of cigar users (Supplementary Table 3, https://stacks.cdc.gov/view/cdc/134702).

### Tobacco Product Use Over Time

From 2022 to 2023, among high school students, statistically significant declines (p<0.05) occurred in current use of any tobacco product (from 16.5% to 12.6%), e-cigarettes (from 14.1% to 10.0%), cigars (from 2.8% to 1.8%), and any combustible tobacco product (from 5.2% to 3.9%). Among middle school students, statistically significant increases (p<0.05) occurred in current use of any tobacco product (from 4.5% to 6.6%) and multiple tobacco products (from 1.5% to 2.5%). Among middle school and high school students combined, no significant change in current use of any composite measure or individual tobacco product was observed.

## Discussion

Current use of any tobacco product by high school students declined by an estimated 540,000 students, from 2.51 million in 2022 (*3*) to 1.97 million in 2023. In 2023, 22.2% of middle and high school students (representing 6.21 million) reported ever using any tobacco product, and 10.0% of students (representing 2.80 million) reported current use of any tobacco product. Similar to 2022 (*3*), ever use of any tobacco product was lowest among non-Hispanic Asian students and did not differ significantly across most racial and ethnic groups.

E-cigarettes have been the most commonly used tobacco product among U.S. youths since 2014 (*4*). Youth e-cigarette use is a critical public health concern, because approximately one half of students ever using e-cigarettes reported using them currently, indicating that many young persons who try e-cigarettes remain e-cigarette users. In 2023, 10.0% of high school students and 4.6% of middle school students used e-cigarettes during the past 30 days. From 2022 (*3*) to 2023, a significant decline in current e-cigarette use occurred among high school students (from 14.1% to 10.0%), while no statistically significant change occurred among middle school students (from 3.3% in 2022 to 4.6% in 2023). The decline since 2022 in high school student e-cigarette use is likely attributable to multiple factors, such as ongoing efforts at the national, state, and local levels to implement tobacco control strategies, including Food and Drug Administration (FDA) regulatory actions. Continued surveillance is needed to determine the trajectory of middle school e-cigarette use. Despite the decline in e-cigarette use among high school students, close to 40% of high school students using e-cigarettes reported frequent use, and 29.9% reported daily use. Furthermore, 550,000 middle school students currently used e-cigarettes, including 20.7% reporting frequent use. Similar patterns were observed in 2022 for both middle school and high school students. These findings are concerning, because adolescents have reported symptoms of nicotine dependence when using tobacco products only 1–3 days per month (*1*). Efforts aimed at reducing nicotine dependence among adolescents by preventing initiation of tobacco products is important (*5*).

Among students who reported current e-cigarette use, disposables were the most commonly used device type. Disposable e-cigarettes have been gaining market share; they are relatively inexpensive, have a high nicotine content, and are available in flavors appealing to youths (e.g., fruit and candy) (*6*). In January 2020, FDA announced that it would prioritize enforcement against prefilled e-cigarettes in flavors other than tobacco and menthol (*7*). In 2023, NYTS for the first time assessed tobacco-flavored product use, use of flavors that included the word “ice” or “iced” in their name, and use of concept flavors. These results, combined with results of other flavored tobacco product use research, continue to demonstrate the high appeal of flavored tobacco products among young persons.

Multiple factors continue to influence tobacco product use and initiation among middle and high school students, including availability of flavored products, marketing, and misperceptions regarding harm. Continued surveillance provides an understanding of the prevalence and frequency of tobacco product use, the popularity of specific brands and flavors, and how product use behaviors change over time as the tobacco product marketplace continues to diversify.

### Limitations

The findings in this report are subject to at least three limitations. First, data were obtained by self-report, which can result in social desirability and recall biases, although previous research suggests that self-reported measures of tobacco use among persons aged 12–21 years correlate with biomarkers of tobacco use (*8*). Second, these findings might not be generalizable to youths who are home-schooled, have dropped out of school, are in detention centers, or are enrolled in alternative schools. Finally, the response rate for the 2023 NYTS was lower than that for the 2022 NYTS (30.5% in 2023 versus 45.2% in 2022). The lower response rate can increase the potential for bias and result in higher SEs for some estimates; higher SEs can reduce the power to detect a significant difference, if there is one, when doing year to year comparisons, especially for certain population groups. Adjustments were made to the survey weights to reduce the potential for nonresponse bias. Therefore, 2023 NYTS estimates may be compared with 2022 NYTS estimates for the same population groups.

### Implications for Public Health Practice

In 2023, 10.0% (representing 2.80 million) of U.S. middle and high school students reported current tobacco product use. A significant decline in current e-cigarette use occurred among high school students from 2022 to 2023 (from 14.1% to 10.0%). Given the negative health consequences of tobacco use (*2*) and the unique harms associated with adolescent nicotine exposure (*1*), prevention of tobacco use by youths is imperative. Thus, a continued comprehensive approach to tobacco use prevention is needed to further reduce tobacco use among youths, based on knowledge about youth product use behaviors. Further, longstanding and proven tobacco prevention policies, such as price increases, comprehensive smoke-free policies (that include e-cigarettes), counter-marketing campaigns, and health care intervention, will continue to reduce youth initiation and tobacco use (*5*).

## Footnotes

* The term “tobacco” as used in this report refers to commercial tobacco products and not to sacred and traditional use of tobacco by some American Indian communities.

† Data were weighted to account for complex survey design and to adjust for nonresponse. The weighted proportions of students in each grade matched national population proportions for U.S. public and private schools derived from data from Market Data Retrieval Inc. 2021–2022 Common Core of Data and the National Center for Education Statistics 2019–2020 Private School Universe Study. Population total estimates were rounded down to the nearest 10,000 persons.

§ Products include e-cigarettes, cigarettes, cigars (cigars, cigarillos, or little cigars), smokeless tobacco (chewing tobacco, snuff, dip, or snus), hookahs, heated tobacco products, nicotine pouches, pipe tobacco, bidis (small brown cigarettes wrapped in a leaf), and other oral nicotine products (lozenges, discs, tablets, gums, dissolvable tobacco products, and other products). In 2023, dissolvable tobacco products were reclassified from smokeless tobacco to other oral nicotine products.

¶ Small, flavored pouches contain nicotine that comes from tobacco. Users place them in their mouth. Nicotine pouches are different from other smokeless tobacco products such as snus, dip, or chewing tobacco, because they do not contain any tobacco leaf.

** Heated tobacco products heat processed tobacco leaf in the form of sticks (“heatsticks”), plugs, or capsules to produce a vapor that the user inhales. They are different from e-cigarettes, which heat a liquid to produce a vapor.

†† Any tobacco product use was defined as use of one or more of the following tobacco products: e-cigarettes, cigarettes, cigars, smokeless tobacco, hookahs, heated tobacco products, nicotine pouches, pipe tobacco, bidis, or other oral nicotine products.

§§ Any combustible tobacco product use is defined as use of one or more of the following tobacco products: cigarettes, cigars, hookahs, pipe tobacco, or bidis.

¶¶ Multiple tobacco product use was defined as use of two or more of the following tobacco products: e-cigarettes, cigarettes, cigars, smokeless tobacco, hookahs, heated tobacco products, nicotine pouches, pipe tobacco, bidis, or other oral nicotine products.

*** Categories used are “disposables,” “prefilled or refillable pods or cartridges,” “tanks or mod system,” or “don't know the type.” Disposable e-cigarettes come prefilled with e-liquid and are designed to be discarded once empty. Some pods or cartridges come prefilled with e-liquid that is replaced after use, although others can be refilled by the user. Tank or mod-type devices can also be refilled but are also usually customizable.

††† Brand response options included blu, Breeze, Elf Bar, Esco Bars, Fume, HQD, JUUL, Kangvape (including Onee Stick), Logic, Mr. Fog, NJOY, SMOK (including NOVO), Suorin (including Air Bar), Vuse, “some other brand(s) not listed here” with space for a write-in name, and “not sure/I don’t know the brand.” Write-in responses corresponding to an original response option were recoded.

§§§ Flavor type was determined by response to the question, “In the past 30 days when you used e-cigarettes, what flavors did you use? (Select one or more).” Those who selected “some other flavor not listed here” could provide a write-in response; write-in responses corresponding to an original response option were recoded.

¶¶¶ 45 C.F.R. part 46.102(l)(2), 21 C.F.R. part 56; 42 U.S.C. Sect. 241(d); 5 U.S.C. Sect. 552a; 44 U.S.C. Sect. 3501 et seq.

**** Estimates among non-Hispanic Asian and non-Hispanic Native Hawaiian or other Pacific Islander students were statistically unreliable for all current measures and are not reported.

†††† Current users were asked, “Did any of the flavors you used in the past 30 days have names or descriptions that included the word ‘ice’ or ‘iced’ (for example, blueberry ice or strawberry ice)?” Those who reported using only “unflavored” e-cigarettes (n = 60) or nicotine pouches (n = 11) were not asked the question.

§§§§ Current users were asked, “Did any of the flavors that you used in the past 30 days have a name that did not describe a specific flavor, such as ‘solar,’ ‘purple,’ ‘jazz,’ ‘island bash,’ ‘fusion,’ or some other word or phrase?” Those who reported using only “unflavored” e-cigarettes (n = 60) or nicotine pouches (n = 11) were not asked the question.
